# A review of extracorporeal membrane oxygenation in child after cardiac surgery: analyses of outcomes

**DOI:** 10.1186/1749-8090-8-S1-O80

**Published:** 2013-09-11

**Authors:** M Asano, Y Nakai, H Matsumae, T Ukai, N Nomura, A Mishima

**Affiliations:** 1 Cardiovascular Surgery, Nagoya City University, Nagoya, Japan

## Background

The use of extracorporeal membrane oxygenation (ECMO) after cardiac surgery in child has continued to increase. However, the survivals have remained less than 50% with a significant rate of complications. We aim to assess the morbidity and mortality in children requiring ECMO after cardiotomy and determine factors affecting outcomes.

## Methods

Sixty-two ECMO operations in 58 children who required post-cardiotomy ECMO between January 2002 and December 2012 were reviewed. The Cox proportional hazards model was used for the univariate and multivariate prognostic risk analyses for the weaning of ECMO or the discharge of hospital.

## Results

Age and weight were 12±17months and 6.4±3.3kg, respectively. Twenty patients had single ventricle and 38 had biventricular physiology. The duration of ECMO was 8.4±4.4 days. Fifty-two (84%) were successfully weaned off ECMO and 34 (55%) survived to hospital discharge. The weaning from ECMO was affected by peak serum lactate during ECMO (risk ratio=1.02, 95%CI: 1.003-1.036, p=0.0181) and the diagnosis of isomerism (46, 0.0004-0.41, 0.0085) by multivariate analysis. Indication for ECMO and surgical procedures were not significant predictors. Factors associate with failure of hospital discharge despite successful decannulation were as follows: weight (0.32, 0.14-0.62, 0.0001), ECMO duration (1.3, 1.02-1.72, 0.0352), the duration of the day between decannulation and the first day of negative water balance after ECMO (1.3, 1.13-1.54, 0.0001), the use of nitric oxide gas after ECMO (8.4, 1.81-46.66, 0.0068). Survivor vs. non-survivor to the hospital discharge was 7.1±4.1 vs. 10.0±4.2 (days) in ECMO duration (p=0.0073) and 2.1±1.5 vs. 8.3±10.2 (days) in the negative water balance (p=0.0004).

## Conclusions

Higher lactate levels and isomerism are significant factors associated with mortality during ECMO. Unable to obtain earlier negative balance and the inhalation of nitric oxide increase risk of death after successful decannulation.

